# Crystal structure of peroxiredoxin 3 from *Vibrio vulnificus* and its implications for scavenging peroxides and nitric oxide

**DOI:** 10.1107/S205225251701750X

**Published:** 2018-01-01

**Authors:** Jinsook Ahn, Kyung Ku Jang, Inseong Jo, Hasan Nurhasni, Jong Gyu Lim, Jin-Wook Yoo, Sang Ho Choi, Nam-Chul Ha

**Affiliations:** a Department of Agricultural Biotechnology, Seoul National University, 1 Gwanak-ro, Seoul 08826, Republic of Korea; b College of Pharmacy, Pusan National University, Busandaehak-ro, Pusan 46241, Republic of Korea

**Keywords:** nitric oxide, peroxiredoxins, crystal structure, *Vibrio vulnificus*, protein structure, X-ray crystallography

## Abstract

Peroxiredoxins (Prxs) are ubiquitous cysteine-based peroxidase enzymes. Recently, a new type of Prx, *Vv*Prx3, was identified in the pathogenic bacterium *Vibrio vulnificus* as being important for survival in macrophages. It employs only one catalytic cysteine residue to decompose peroxides. Here, crystal structures of *Vv*Prx3 representing its reduced and oxidized states have been determined, together with an H_2_O_2_-bound structure, at high resolution. The crystal structure representing the reduced Prx3 showed a typical dimeric interface, called the A-type interface. However, *Vv*Prx3 forms an oligomeric interface mediated by a disulfide bond between two catalytic cysteine residues from two adjacent dimers, which differs from the doughnut-like oligomers that appear in most Prxs. Subsequent biochemical studies showed that this disulfide bond was induced by treatment with nitric oxide (NO) as well as with peroxides. Consistently, NO treatment induced expression of the *prx3* gene in *V. vulnificus*, and *Vv*Prx3 was crucial for the survival of bacteria in the presence of NO. Taken together, the function and mechanism of *Vv*Prx3 in scavenging peroxides and NO stress *via* oligomerization are proposed. These findings contribute to the understanding of the diverse functions of Prxs during pathogenic processes at the molecular level.

## Abstract 2

The crystal structure of a bacterial 1-Cys peroxiredoxin has been solved, revealing an oligomeric interface.

## 1. Introduction   

Most organisms have evolved efficient defence systems that rapidly detoxify potentially damaging reactive oxygen species (ROS). ROS are generated during aerobic respiration and in the host immune system (Temple *et al.*, 2005 ▸; Cabiscol *et al.*, 2000 ▸; Halliwell, 2006 ▸). Peroxiredoxins (Prxs) represent a superfamily of thiol-specific antioxidant peroxidases that use an invariant cysteine residue(s) (Engelman *et al.*, 2013 ▸). They are highly conserved throughout evolution and are ubiquitously found from humans to bacteria. Prxs decompose peroxides such as hydrogen peroxide (H_2_O_2_) and alkyl hydroperoxides in the cytosol (Rhee *et al.*, 2005 ▸; Wood *et al.*, 2003 ▸; Engelman *et al.*, 2013 ▸). Recent research has reported that Prxs are also involved in the detoxification of reactive nitrogen species (RNS), which are produced in phagocytes as a key component for killing invading bacteria (Wong *et al.*, 2002 ▸; Bryk *et al.*, 2000 ▸). Prxs have been found to detoxify peroxi­nitrite (ONOO^−^) and nitrosylated thiols (Fang, 2004 ▸; Beckman & Koppenol, 1996 ▸; Pfeiffer *et al.*, 1997 ▸).

Prxs have a peroxidatic cysteine (C_P_) that reacts with peroxides in the first step of catalysis. The C_P_ is oxidized to cysteine sulfenic acid (C_P_-SOH) by attacking the O—O bond of peroxides during the peroxidase reaction, resulting in the decomposition of peroxides (Wood *et al.*, 2003 ▸). The C_P_-SOH is then reduced during the resolution step, which is needed to recycle the protein. Depending on the resolution step, Prxs are classified into three types, referred to as typical 2-Cys, atypical 2-Cys and 1-Cys Prxs (Hall *et al.*, 2011 ▸). Both types of 2-Cys Prx have an additional conserved cysteine residue called the resolving cysteine (C_R_), which forms a stable intermolecular or intramolecular disulfide bond with the C_P_-SOH. While C_R_ is located in a separate subunit in typical 2-Cys Prxs, it is located within the same subunit in atypical 2-Cys Prxs (Seo *et al.*, 2000 ▸; Hall *et al.*, 2009 ▸). The disulfide bond in 2-Cys Prxs can be broken into two free thiols by cellular reductants (Chae *et al.*, 1999 ▸). In contrast, the C_P_-SOH of 1-Cys Prxs has been implicated to be directly reduced by cellular reductants such as glutaredoxin (Grx) and/or the reduced form of glutathione (GSH) (Noguera-Mazon *et al.*, 2006 ▸; Kang *et al.*, 1998 ▸; Rhee *et al.*, 2005 ▸). In a yeast 1-Cys Prx, the C_P_-SOH has been proposed to form a disulfide bond with the C_P_ residue of another molecule of Prx (Pedrajas *et al.*, 2016 ▸).

Although the oligomeric states of Prxs differ depending on the type of Prx, most oligomeric Prxs are formed *via* two major distinct interfaces: B-type and A-type interfaces (Parsonage *et al.*, 2005 ▸). In typical 2-Cys Prxs, dimers are primarily formed *via* the B-type interface, in which C_P_ and C_R_ are located in different protomers and disulfide formation between C_P_ and C_R_ is facilitated by peroxides (Echalier *et al.*, 2005 ▸). In most 2-Cys Prxs, five dimers are further associated *via* the A-type interface to form a doughnut-like decamer (Wood *et al.*, 2002 ▸; Parsonage *et al.*, 2005 ▸). Most atypical 2-Cys Prxs and 1-Cys Prxs exhibit dimeric assembly *via* the A-type interface (Evrard *et al.*, 2004 ▸; Li *et al.*, 2005 ▸). Exceptionally, a non-A and non-B oligomeric interface between C_P_ and C_R_ was observed in human Prx5 (*Hs*Prx5), which belongs to the atypical 2-Cys Prx family (Smeets *et al.*, 2008 ▸) and showed redox-dependent structural changes in the C_P_-containing region. The structural change was distinguished from that in some typical 2-Cys Prxs (Wood *et al.*, 2003 ▸; Parsonage *et al.*, 2005 ▸). In the presence of excess peroxides the C_P_ can be overoxidized to cysteine sulfinic acid or cysteine sulfonic acid (C_P_-SO_2_H or C_P_-SO_3_H, respectively). Interestingly, the protomers with overoxidized C_P_ exhibit the same conformation as in Prxs in the reduced state (Mizohata *et al.*, 2005 ▸; Nakamura *et al.*, 2006 ▸).

*Vibrio vulnificus* is a highly virulent foodborne pathogen that can cause fatal septicaemia (Oliver, 2005 ▸; Horseman & Surani, 2011 ▸). The bacterium has three different types of Prx: Prx1, Prx2 and Prx3. Prx1 and Prx2 belong to the typical 2-Cys Prx family of proteins and their expression is controlled by the H_2_O_2_-activated transcription factor OxyR (Kim *et al.*, 2014 ▸; Jo *et al.*, 2015 ▸). *V. vulnificus* Prx3 (*Vv*Prx3) belongs to the 1-Cys class of Prxs, with one conserved catalytic cysteine (Cys48), and has been shown to be involved in pathogenicity in a mouse model. According to the PeroxiRedoxin Classification Index (PREX) database (http://www.csb.wfu.edu/prex), it belongs to the Prx5 subfamily. *Vv*Prx3 expression is controlled by IscR, which is activated by oxidative stress and iron starvation (Lim *et al.*, 2014 ▸). In this study, we determined crystal structures of *Vv*Prx3 in two different forms and in complex with H_2_O_2_. Based on the crystal structures, we present a molecular mechanism of 1-Cys Prxs that highlights the oligomer interface containing a disulfide bond between two peroxidatic cysteine residues. More importantly, we examined the expression of *prx3* in *V. vulnificus* induced by nitric oxide gas (NO); consistently, *Vv*Prx3 reacts with nitric oxide.

## 2. Experimental procedures   

### 2.1. Plasmid construction   

The *Vv*Prx3 (C48D/C73S) and *Vv*Prx3 (C73S) genes of *V. vulnificus* were cloned into the pET-21c vector (Invitrogen). To clone the *Vv*Prx3 gene for the expression of *Vv*Prx3 (C73S), two primers (forward, 5′-GGTCATATGATCGCTCAAGGCCAAACTTTACC-3′; reverse, 5′-CCTCTCGAGCGCGGCAAGAATCGTTTCAGC-3′) were designed, which contained NdeI and XhoI sites (underlined), respectively. Site-directed mutagenesis was performed in two subsequent PCR reactions for the expression of *Vv*Prx3 (C48D/C73S) (Ho *et al.*, 1989 ▸). The PCR products and pET-21c plasmid were digested by NdeI and XhoI, and the digested products were ligated with DNA ligase. The ligation products were transformed into *Escherichia coli* C43 (DE3) cells by heat shock. Finally, the recombinant plasmid was confirmed by DNA sequencing.

### 2.2. Expression and purification of recombinant proteins   

Recombinant proteins were expressed in LB medium containing 50 µg ml^−1^ ampicillin, with growth at 310 K to an OD_600_ of 0.5. Samples were induced with 0.5 m*M* isopropyl β-d-1-thiogalactopyranoside (IPTG) for 6 h at 303 K. The cells were harvested and resuspended in lysis buffer consisting of 20 m*M* Tris–HCl pH 8.0, 0.15 *M* NaCl, 2 m*M* β-mercapto­ethanol. After disrupting the cells with a French press, the cell debris was removed by centrifugation. The supernatant was loaded onto Ni–NTA affinity resin (Qiagen, The Netherlands) that had been pre-incubated with lysis buffer. The target protein was eluted with lysis buffer supplemented with 250 m*M* imidazole. Eluted recombinant proteins were diluted threefold with 20 m*M* Tris–HCl pH 8.0 buffer containing 2 m*M* β-mercaptoethanol and loaded onto a HiTrap Q column (GE Healthcare, USA). A linear gradient of increasing NaCl concentration was applied to the column. The fractions containing the protein were pooled, concentrated and applied onto a size-exclusion chromatography column (HiLoad 16/600 Superdex 200 pg, GE Healthcare) pre-equilibrated with lysis buffer. The final protein samples were concentrated to 20 mg ml^−1^ using a centrifugal filter concentration device (Millipore; 10 kDa cutoff) and stored frozen at 193 K until use.

### 2.3. Crystallization, structure determination and refinement   

Crystallization was performed at 287 K using the hanging-drop vapour-diffusion method. Crystals of Prx3 (C48D/C73S) were obtained using a precipitant solution consisting of 0.2 *M* NaCl, 0.8 *M* sodium citrate, 0.1 *M* Tris–HCl pH 6.5. The structure of reduced Prx3 (C48D/C73S) was determined using the molecular-replacement method with *MOLREP* in the *CCP*4 package (Winn *et al.*, 2011 ▸) using a putative thioredoxin reductase from *Burkholderia ceno­cepacia* (PDB entry 4f82; Seattle Structural Genomics Center for Infectious Disease, unpublished work) as a search model. The crystal of Prx3 (C48D/C73S) obtained under reduced conditions [reduced Prx3 (C48D/C73S)] belonged to space group *P*3_2_21, with unit-cell parameters *a* = 73.9, *b* = 73.9, *c* = 62.3 Å and one protein molecule in the asymmetric unit (Table 1 ▸). After 5 d, Prx3 (C73S) crystals appeared with a precipitant solution consisting of 0.1 *M* sodium citrate pH 5.5, 0.2 *M* ammonium acetate, 22%(*w*/*v*) PEG 4000. Crystals were exchanged into the appropriate mother liquor containing 20%(*v*/*v*) glycerol, mounted on cryo-loops and flash-cooled in a liquid-nitrogen stream at 100 K. X-ray diffraction data sets were collected on beamline 5C at the Pohang Accelerator Laboratory and were processed with the *HKL*-2000 package (Otwinowski & Minor, 1997 ▸). The crystal of Prx3 (C73S) obtained under oxidized conditions [oxidized Prx3 (C73S)] belonged to space group *P*2_1_2_1_2_1_, with unit-cell parameters *a* = 39.5, *b* = 57.4, *c* = 124 Å and two protein molecules in the asymmetric unit (Table 1 ▸). The oxidized Prx3 (C73S) crystals were flash-cooled using a crystallization solution with the same cryoprotectant as used for the reduced Prx3 (C48D/C73S) crystals in a nitrogen stream at 100 K. The initial model of oxidized Prx3 (C73S) was determined by molecular replacement using the structure of reduced Prx3 (C48D/C73S) as a model and was refined at 2.0 Å resolution (Table 1 ▸). The final structure of oxidized Prx3 (C73S) was refined at 1.48 Å resolution using *PHENIX* (Afonine *et al.*, 2012 ▸). To obtain H_2_O_2_-bound Prx3 (C48D/C73S) crystals, 20 m*M* H_2_O_2_ was added to the reservoir solution and 500 µ*M* H_2_O_2_ was added to the hanging-drop solution containing the crystals. After 10 d, the H_2_O_2_-bound Prx3 (C48D/C73S) crystals were flash-cooled using the same cryoprotectant as used for the reduced Prx3 (C48D/C73S) crystals in a nitrogen stream at 100 K. The crystals belonged to space group *P*1, with unit-cell parameters *a* = 75.08, *b* = 97.72, *c* = 97.49 Å and 12 protein molecules in the asymmetric unit. Statistics regarding data collection and processing are presented in Table 1 ▸.

### 2.4. Oxidation of *Vv*Prx3 by H_2_O_2_ or NO   

To prepare the reduced *Vv*Prx3 (C73S) protein, 10 m*M* DTT was added to the purified *Vv*Prx3 (C73S) protein and was then removed using a HiPrep 26/10 Desalting column (GE Healthcare, USA) pre-equilibrated with 20 m*M* Tris–HCl pH 8.0 buffer containing 150 m*M* NaCl. The reduced *Vv*Prx3 (C73S) mutant protein was reacted with various concentrations of H_2_O_2_ or tertiary butyl hydrogen peroxide (*t*-BOOH) for 30 min at 298 K in 1 ml 20 m*M* Tris–HCl buffer pH 8.0 containing 150 m*M* NaCl. The reaction mixture was treated with iodoacetamide (IAA) to stop the reaction and subjected to SDS–PAGE under nonreducing or reducing conditions. To analyze Prx3 oxidation during the time course, samples were treated with 50 µ*M* H_2_O_2_ for 0–60 s and the reaction was quenched with 10 m*M* *N*-ethylmaleimide (NEM). For reaction with NO, reduced *Vv*Prx3 (C73S) protein (10 nmol) was treated with 1 mg NO-releasing PLGA–PEI nanoparticles (NO/PPNPs) in 10 ml reaction mixture in 20 m*M* Tris pH 7.5, 150 m*M* NaCl. The amount of NO was calculated based on the release of 3.3 nmol NO per hour by 1 mg NO/PPNPs (Nurhasni *et al.*, 2015 ▸).

### 2.5. Competitive kinetics with horseradish peroxidase (HRP)   

To prepare the reduced *Vv*Prx3 (C73S) protein, the purified protein was treated with 10 m*M* DTT for 30 min at room temperature. After reduction of the protein, residual DTT was removed using a HiPrep 26/10 Desalting (GE Healthcare, USA) column pre-equilibrated with 20 m*M* Tris–HCl pH 7.5 buffer containing 150 m*M* NaCl. Reaction mixtures containing 5 µ*M* horseradish peroxidase (HRP; Sigma–Aldrich) and various concentrations (0–9.15 µ*M*) of reduced *Vv*Prx3 (C73S) were treated with 2.5 µ*M* H_2_O_2_ at room temperature in reaction buffer consisting of 50 m*M* sodium phosphate pH 7.4, 150 m*M* NaCl. The concentration of HRP was calculated using the absorbance at 403 nm (∊_403_ = 1.02 × 10^5^ *M* ^−1^ cm^−1^) and the concentration of *Vv*Prx3 (C73S) was determined at 280 nm (∊_*Vv*Prx3_ = 8.48 × 10^3^ *M* ^−1^ cm^−1^). The competitive kinetics of *Vv*Prx3 (C73S) were determined using a previously described procedure (Ogusucu *et al.*, 2007 ▸; Winterbourn & Peskin, 2016 ▸; Cox *et al.*, 2009 ▸). In brief, the ratio of inhibition of HRP oxidation [*F*/(1 − *F*)] was measured at 403 nm with a spectro­photometer using a 10 mm cuvette. The second-order rate constants (*k*_1_) of *Vv*Prx3 (C73S) were determined from the slope of a plot of [*F*/(1 − *F*)]*k*_HRP_[HRP] against [*Vv*Prx3 (C73S)] (*k*_HRP_ = 1.7 × 10^7^ *M* ^−1^ s^−1^).

### 2.6. Gel-filtration chromatography   

A Superose 6 HR 10/30 column coupled to an FPLC instrument (Biologic Duo Flow, Bio-Rad) was equilibrated with 20 m*M* Tris–HCl pH 8.0, 150 m*M* NaCl. Samples were incubated with 10–30 µ*M* H_2_O_2_ for 30–60 min at room temperature before loading them onto the column to form the intermolecular disulfide bonds of *Vv*Prx3 (C73S). Samples were subjected to chromatography, which was performed at a flow rate of 0.2 ml min^−1^. Several standard preparations were run to calibrate the column: ferritin (440 kDa), aldolase (158 kDa), conalbumin (75 kDa), ovalbumin (43 kDa), carbonic anhydrase (29 kDa) and ribonuclease A (13.7 kDa) (Supplementary Fig. S8). The absorbance at 280 nm was used to monitor the presence of the proteins.

### 2.7. Survival in the presence of nitric oxide (NO)   

The *V. vulnificus* cultures were grown to an *A*_600_ of 0.5 in LBS broth [LB broth supplemented with 2.0%(*w*/*v*) NaCl] at 30°C and the cells were harvested by centrifugation at 1500*g* for 7 min at room temperature. The cell pellets were resuspended in M9 minimal medium supplemented with 0.4%(*w*/*v*) glucose. The bacterial cells [∼4 × 10^7^ colony-forming units (CFU)] were exposed to 0.1%(*w*/*v*) NO/PPNPs (Nurhasni *et al.*, 2015 ▸) for 2 h and an aliquot was acquired every 20 min. The number of live bacterial cells in the aliquots was determined as the CFU on LBS agar plates. Bacterial strains and plasmids are described in Supplementary Table S1.

### 2.8. RNA purification and transcript analysis   

Wild-type *V. vulnificus* cells grown to an *A*_600_ of 0.5 in LBS broth were exposed to various concentrations of PLGA–PEI nanoparticles (PPNPs) and NO/PPNPs for 20 min and were harvested to isolate the total RNA. Total RNAs were isolated using an RNeasy Mini kit (Qiagen). For quantitative real-time PCR (qRT-PCR), the concentration of total RNA from the strains was measured using a NanoVue Plus spectrophoto­meter (GE Healthcare). The cDNA was synthesized from 1 µg total RNA using an iScript cDNA-synthesis kit (Bio-Rad). Real-time PCR amplification of cDNA was performed using a Chromo 4 real-time PCR detection system (Bio-Rad) with a pair of specific primers (Supplementary Table S2) as described previously (Jang *et al.*, 2016 ▸). The relative expression levels of *iscR* and *prx3* mRNA in the same amount of total RNA were calculated using the 16S rRNA expression level as an internal reference for normalization.

## 3. Results   

### 3.1. Structural determination of reduced Prx3 (C48D/C73S), oxidized Prx3 (C73S) and H_2_O_2_-bound Prx3 (C48D/C73S)   

*Vv*Prx3 contains two cysteine residues: Cys48 and Cys73. Cys48 is the C_P_ residue that is crucial for the function of the protein. However, Cys73 is not involved in protein function because the enzymatic activity of *Vv*Prx3 (C73S) was similar to that of wild-type *Vv*Prx3 in the GSH/Grx3/GR system (Lim *et al.*, 2014 ▸). To focus on the function of Cys48 and prevent unwanted disulfide-bond formation at Cys73 during SDS–PAGE analyses, Cys73 was replaced with a serine residue in this study. To investigate an oxidized structure of Prx3 containing a disulfide bond, the crystal of the oxidized *Vv*Prx3 (C73S) protein was obtained under crystallization conditions without a reducing agent. The crystal structure of *Vv*Prx3 (C73S) [named oxidized Prx (C73S) in this study] was determined at 1.5 Å resolution in space group *P*2_1_2_1_2_1_ using the molecular replacement method. The resulting structure was refined to an *R*_work_ of 0.181 and an *R*_free_ of 0.198 (Table 1 ▸). The final model comprises residues 1–157 and contains two protein molecules in the asymmetric unit (Table 1 ▸). To study the structure of Prx3 in the reduced state, crystals of a *Vv*Prx3 (C48D/C73S) variant were also obtained. The C_P_ Cys48 in *Vv*Prx3 was replaced with a non-oxidizable Asp residue, which could also partly mimic an over-oxidized form (Cys-SO_2_H) of *Vv*Prx3 (Jo *et al.*, 2015 ▸). The crystal belonged to a different space group, *P*3_2_21, and the *Vv*Prx3 (C48D/C73S) structure [named reduced Prx (C48D/C73S) in this study] was determined by the molecular replacement approach. The final model, refined at 1.9 Å resolution (*R*_work_ = 0.220 and *R*_free_ = 0.258), contains one protomer (residues 1–18 and residues 22–157) in the asymmetric unit (Table 1 ▸). To examine the interactions between *Vv*Prx3 and peroxides, the H_2_O_2_-bound Prx3 (C48D/C73S) structure was determined at 1.9 Å resolution. A high concentration (500 µ*M*) of H_2_O_2_ was incubated with crystals of reduced Prx3 (C48D/C73S), which changed the space group and unit-cell parameters. The resulting structure was refined to an *R*_work_ of 0.195 and an *R*_free_ of 0.244. Electron-density maps were well defined for all 12 protomers in the asymmetric unit (Table 1 ▸).

**Figure 1:**
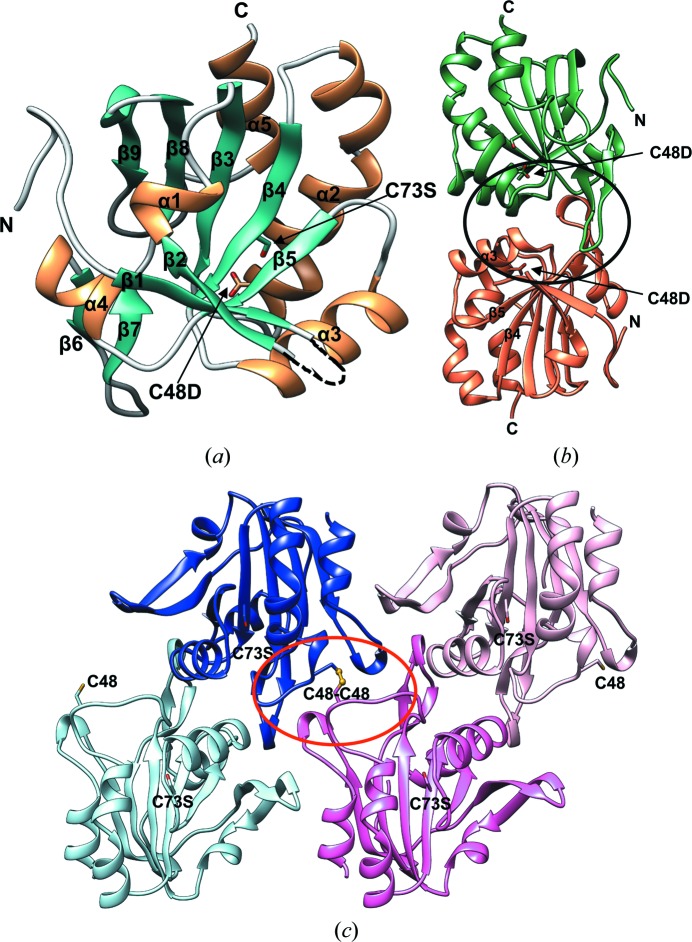
Overall structure and oligomeric interfaces of *Vv*Prx3. (*a*) Ribbon diagram of the reduced Prx3 (C48D/C73S) protomer; α-helices, β-strands and loops are represented in gold, green and grey, respectively. The black arrows indicate the side chains of the mutated residues Cys48 and Cys73. (*b*) Dimeric structure of reduced Prx3 (C48D/C73S) formed by an A-type interface (black circle). The two protomers are coloured differently (green and salmon). The side chains of Cys48 and Cys73 are shown in ball-and-stick representation. The black arrows indicate the side chains of the mutated residues (C48D). (*c*) The asymmetric unit of oxidized Prx3 (C73S) with intact Cys48. Two Cys48 residues from adjacent protomers form a disulfide bond. The crystals of oxidized Prx3 (C73S) were grown without reducing agent. Four protomers are drawn in different colours and the side chains of Cys48 and Ser73 are displayed as ball-and-stick models. The red circle represents the C-type interface.

All three crystal structures of *Vv*Prx3 revealed a protomer exhibiting a compact and spherical structure without a C-terminal extension region, similar to the previously reported 1-Cys Prx AhpE from *Mycobacterium tuberculosis* (Supplementary Figs. S1 and S2; Li *et al.*, 2005 ▸). The *Vv*Prx3 protomer is formed based on a central five-stranded β-sheet (β5–β4–β3–β8–β9; Fig. 1 ▸ *a*). α-Helices α2 and α5 are located on one side of the central β-sheet, while α-helix α4 and the β-hairpin (β1 and β2) are on the other side of the central β-sheet. Strands β4–β3–β8–β9 and helices α2, α4 and α5 comprise the typical Trx fold. In the peripheral region, a β4–α3–β5 structural motif is located in the A-type dimeric interface (Figs. 1 ▸ *a* and 1 ▸ *b*). C_P_ Cys48 (or the mutated Asp48) is located at the N-terminus of the long, kinked α2, similar to other Prxs. The mutated Ser73 is buried in the Trx fold region (Fig. 1 ▸ *a*). Structural superposition of reduced Prx (C48D/C73S) on human PrxV (PDB entry 1oc3; sequence identity 52%; Evrard *et al.*, 2004 ▸) revealed high structural similarity, with an r.m.s.d. of 1.46 Å between the C^α^ positions of 247 matched amino acids (Supplementary Fig. S2).

### 3.2. Comparison of structures of *Vv*Prx3   

**Figure 2:**
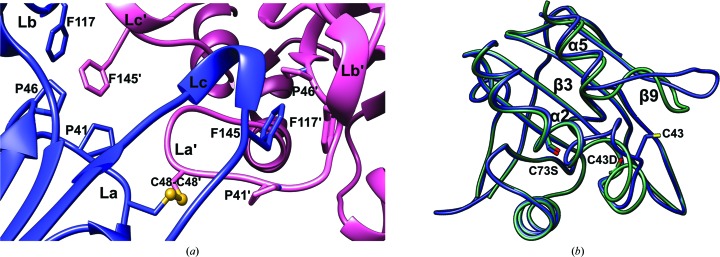
Structural comparison of *Vv*Prx3 in reduced and oxidized states. (*a*) A magnified view focusing on the interactions at the C-type interface (Fig. 1 ▸ *c*; red circle) with the intermolecular disulfide bond present between Cys48 residues. The side chains involved in the hydrophobic core are shown in ball-and-stick representation. The loops in this interface are labelled La, Lb and Lc, indicating the loop connecting β3 and α2, the loop connecting β6 and β7, and the loop connecting β9 and α5, respectively. Primes are used in the labels to distinguish the protomers. (*b*) Structural superposition of *Vv*Prx3 in the reduced form (green) and the disulfide-bridged (oxidized) form (blue).

All three crystal structures of *Vv*Prx3 [reduced Prx3 (C48D/C73S), oxidized Prx3 (C73S) and H_2_O_2_-bound Prx3 (C48D/C73S)] showed a similar dimeric assembly in the crystals characterized by an A-type dimeric interface, similar to that in AhpE from *M. tuberculosis* (Supplementary Figs. S1 and S2; Li *et al.*, 2005 ▸). In the reduced Prx3 (C48D/C73S) structure the C_p_-containing α2 helix at the active site adopted the ‘fully folded’ or reduced conformation usually observed in Prx structures in the reduced and overoxidized states (Fig. 2 ▸; Mizohata *et al.*, 2005 ▸; Schröder *et al.*, 2000 ▸).

Strikingly, a dimeric interface was found in the oxidized Prx3 (C73S) that was distinct from both the A-type and B-type interfaces (Figs. 1 ▸ *a* and 1 ▸ *b*). This dimeric interface was similar to an exceptional oligomeric interface observed in the oxidized structure of *Hs*Prx5, although this structure contains the disulfide bond between C_P_ and C_R_ (Smeets *et al.*, 2008 ▸), while *Vv*Prx3 directly forms a dimeric interface through the intermolecular disulfide bond between the C_P_ Cys48 residues. This interface was designated the ‘C-type’ interface in this study since these oligomeric interfaces was induced by the disulfide bonds.

The C-type interface mainly consists of two C_P_-containing α2 helices from adjacent protomers (Fig. 1 ▸ *c*). Hydrophobic interactions were observed in the C-type interface that appear to further stabilize this dimeric interface. The Phe145 residue in loop β9–α5 of one protomer forms a hydrophobic core with Phe117 in loop β6–β7, while Pro41 and Pro46 form a hydrophobic core in the loop connecting β3 and α2 of the other molecule (Fig. 2 ▸ *a*). Owing to disulfide bonds and hydrophobic interactions, a significant conformational alteration was observed in the active site of helix α2 containing C_P_ and nearby loops. In addition, loop β9–α5 moved outwards from the core and loop β3–α2 became ordered in the oxidized Prx3 (C73S) structure representing the oxidized structure, compared with the reduced Prx3 (C48D/C73S) structure representing the overoxidized or reduced conformation (Fig. 2 ▸ *b*). In *Hs*Prx5, the conformational change of loop β7–α6 corresponding to loop β9–α5 of *Vv*Prx3 was induced by forming an intramolecular disulfide bond depending on the redox state (Supplementary Fig. S3).

### 3.3. Intermolecular disulfide formation of *Vv*Prx3 reacted with peroxides, H_2_O_2_ and *t*-BOOH   

**Figure 3:**
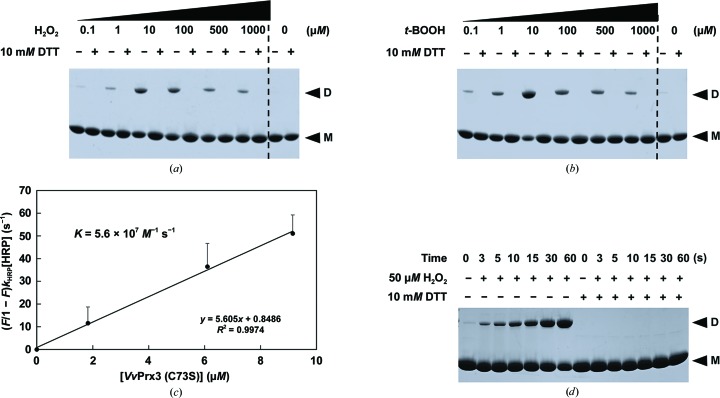
Intermolecular disulfide-bond formation in *Vv*Prx3 (C73S) on treatment with peroxide. Proteins were treated with H_2_O_2_ (*a*) or *tert*-butyl hydrogen peroxide (*t*-BOOH) (*b*) at the concentrations shown for 30 min and the reactions were stopped by adding iodoacetamide. Samples were boiled in the presence (+) or absence (−) of 10 m*M* DTT for SDS–PAGE analysis. (*c*) Determination of the second-order reaction rate constant for the reaction of *Vv*Prx3 and H_2_O_2_ using competition kinetics with HRP. The fitting line was calculated by the least-squares method and the error bars reflect the standard deviation from three independent experiments. (*d*) Purified *Vv*Prx3 (C73S) (70 µ*M*) was reacted with 50 µ*M* H_2_O_2_ for the given times and analyzed by SDS–PAGE. At a time of 30 s, ∼30 µ*M* protein was linked by a disulfide bond as estimated using the density-measuring program *ImageJ*. The protein band for disulfide bond-containing *Vv*Prx3 (D) and bands that lack a disulfide bond (M) are indicated.

To confirm whether the observed disulfide bond in the crystal structure is generated in the solution state, we performed the following biochemical experiment with H_2_O_2_ and *t*-BOOH, which are known substrates of *Vv*Prx3 (Dubbs & Mongkolsuk, 2007 ▸; Beckman & Koppenol, 1996 ▸; Lim *et al.*, 2014 ▸). Reduced *Vv*Prx3 (C73S) protein was incubated with H_2_O_2_ or *t*-BOOH and analyzed by SDS–PAGE under non­reducing conditions to observe intermolecular disulfide-bond formation. While boiling the protein sample for SDS–PAGE, free cysteine residues appeared to form nonspecific disulfide bonds. To avoid these nonspecific disulfide bonds, the free thiol-specific alkylating agent iodoacetamide was added to the sample prior to boiling to block free cysteines. We observed that the protein bands were up-shifted to a dimeric size on the SDS–polyacrylamide gels on treatment with H_2_O_2_ or *t*-BOOH (Figs. 3 ▸ *a* and 3 ▸ *b*). The protein bands shifted back to a monomeric size when a reducing agent was added to cleave disulfide bonds during boiling. These results indicate that two *Vv*Prx3 protomers were linked by an intermolecular disulfide bond between two Cys48 residues. Interestingly, the intensities of the dimeric protein bands decreased at higher concentrations of peroxide. Maximum band intensities were shown at 10 µ*M* H_2_O_2_ or *t*-BOOH (Figs. 3 ▸ *a* and 3 ▸ *b*), likely owing to overoxidation of C_P_ at high peroxide concentrations (Claiborne *et al.*, 1999 ▸; Rhee *et al.*, 2007 ▸). These findings indicate that the intermolecular disulfide bond between C_P_ residues is induced by the peroxides H_2_O_2_ and *t*-BOOH, unlike the prevailing mechanisms for 1-Cys Prxs *via* the direct reduction of C_P_-SOH by Grx or GSH (Noguera-Mazon *et al.*, 2006 ▸; Kang *et al.*, 1998 ▸; Rhee *et al.*, 2005 ▸).

The second-order reaction rate constant (*k*_1_) for H_2_O_2_ consumption by *Vv*Prx3 was measured by employing competition kinetics with horseradish peroxidase (HRP; Winterbourn & Peskin, 2016 ▸; Cox *et al.*, 2009 ▸; Ogusucu *et al.*, 2007 ▸). The measured value was 5.6 × 10^7^ *M* ^−1^ s^−1^, which was of the same order as that for human Prx2 (Peskin *et al.*, 2007 ▸; Fig. 3 ▸ *c*). This finding indicates that *Vv*Prx3 is as efficient as 2-Cys Prxs. To estimate the reaction rate of dimerization of *Vv*Prx3, the amount of dimeric *Vv*Prx3 was measured using the same SDS–PAGE method with different incubation times of *Vv*Prx3 and H_2_O_2_. We observed that almost half of the *Vv*Prx3 formed an intermolecular disulfide bond within 30 s (Fig. 3 ▸ *d*), indicating that the reaction velocity for disulfide-bond formation between *Vv*Prx3-SOH and *Vv*Prx3-SH is comparable to a similar reaction with human Prx2 or Prx3 (Gupta & Carroll, 2014 ▸). Taken together, our findings suggest that dimerization of *Vv*Prx3 could provide a rapid kinetic path to resolving C_P_ and is central to the catalysis of the enzyme.

### 3.4. Redox-dependent oligomerization of *Vv*Prx3   

A protein oligomer representing the oxidized state was found in the oxidized Prx3 (C73S) crystals (Fig. 1 ▸ *c*). The oligomer is generated in a linear arrangement by alternating connections between the protomers *via* A-type contacts and C-type contacts (Supplementary Fig. S4). As observed in Fig. 1 ▸(*c*), peroxides induce a disulfide bond between protomers through the C-type interface. The A-type dimer in the reduced state is further linked by the disulfide bond between the C_P_ residues at the C-type interface, which can lead to the linear oligomer observed in oxidized Prx3 (C73S) crystals.

**Figure 4:**
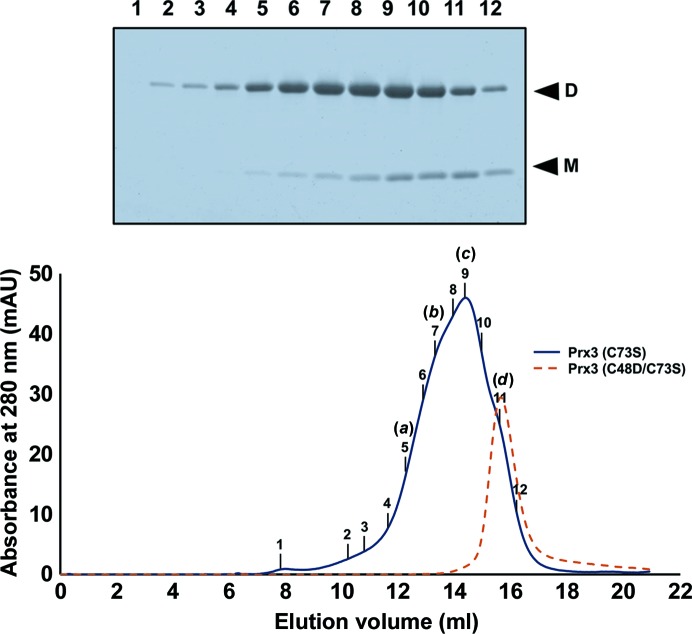
Oligomerization states of *Vv*Prx3 in solution were analyzed using size-exclusion chromatography, and fractions were further analyzed by SDS–PAGE under nonreducing conditions. The *Vv*Prx3 (C73S) protein (33 µ*M*) was incubated with H_2_O_2_ (15 µ*M*) for 60 min before applying size-exclusion chromatography. The result of the native gel electrophoresis is provided in Supplementary Fig. S5. The molecular size was calculated from the standard plot in Supplementary Fig. S8. The molecular sizes of the fractions labelled (*a*)–(*d*) correspond to decamer (∼244 kDa), hexamer (∼143 kDa), tetramer (∼81 kDa) and dimer (∼42 kDa), respectively. The protein band of disulfide-bond-containing *Vv*Prx3 (D) and bands that lack a disulfide bond (M) are indicated. As a control for the non-oxidized sample, *Vv*Prx3 (C73S/C48S) was analyzed using the same column (shown by a broken line).

To investigate changes in the oligomeric states of *Vv*Prx3 proteins induced by oxidative stress, the oligomeric state and disulfide-bond formation of *Vv*Prx3 proteins in the presence/absence of H_2_O_2_ was analyzed by combining size-exclusion chromatography and SDS–PAGE. In these experiments, two Prx variants were used: C73S and C48D/C73S. To create a disulfide bond between the C_P_ residues, *Vv*Prx3 (C73S) protein was treated with H_2_O_2_ before loading it onto a size-exclusion chromatograpy column pre-equilibrated with lysis buffer devoid of reducing agent. The elution profile showed a broad early-eluting peak and shoulders before achieving the dimeric size of *Vv*Prx3, reflecting mixed forms of higher oligomers. Decamers, hexamers, tetramers and dimers were observed on the size-exclusion chromatography column when the molecular sizes were estimated based on the elution volume. The higher oligomeric forms in solution were further confirmed by native gel electrophoresis (Supplementary Fig. S5). The higher oligomeric forms contained more *Vv*Prx3 dimers that are linked by a disulfide bond between two C_P_ residues, as judged by SDS–PAGE under nonreducing conditions (Fig. 4 ▸). These results indicate that the higher oligomeric forms of *Vv*Prx3 were created by noncovalently associated A-type interfaces and disulfide-linked C-type interfaces, as observed in crystals of oxidized Prx3 (C73S).

### 3.5. H_2_O_2_ binding site   

**Figure 5:**
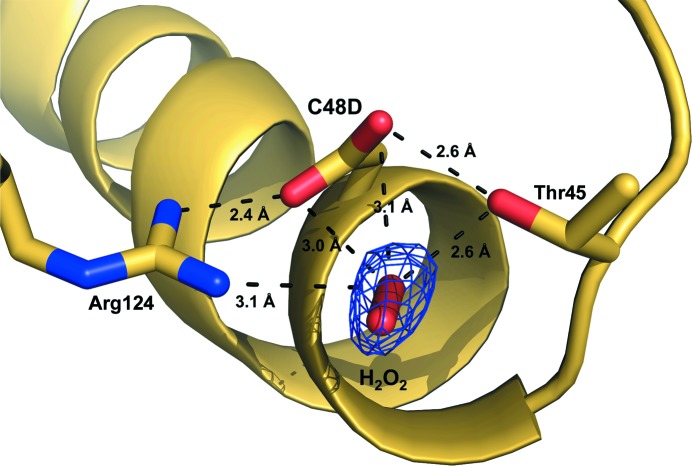
The H_2_O_2_ binding site in H_2_O_2_-bound Prx3 (C48D/C73S) mimics the overoxidized structure. Bound H_2_O_2_ and the residues involved are shown in stick representation at a resolution of 1.9 Å. Broken lines indicate the interactions between residues and H_2_O_2_. The ovoid-shaped electron density indicates H_2_O_2_.

Ovoid-shaped electron density indicating H_2_O_2_ was found near Asp48 and the active-site region in the H_2_O_2_-bound Prx3 (C48D/C73S) structure (Fig. 5 ▸ and Supplementary Fig. S6). The H_2_O_2_ binding site is fully accessible from the solvent since it is exposed to the external medium. The structure is analogous to that of the H_2_O_2_-bound archaeal 2-Cys Prx thio­redoxin peroxidase from *Aeropyrum pernix* K1 (Nakamura *et al.*, 2010 ▸), in which the corresponding cysteine residue was overoxidized and shares the H_2_O_2_ binding site (Supplementary Fig. S6*d*). Thus, it is likely that substrate binding near C_P_ is important in the rapid catalysis of *Vv*Prx3. In Prx structures only one O atom of H_2_O_2_ near Cys48 (or Asp48 in our structure) makes further interactions with *Vv*Prx3, while the other O atom makess no interaction with H_2_O_2_-bound Prx3 (C48D/C73S) (Fig. 5 ▸). Both O atoms in H_2_O_2_ were extensively involved in polar interactions in OxyR, which can recognize only H_2_O_2_ and not alkyl hydrogen peroxides (Jo *et al.*, 2015 ▸). This difference in H_2_O_2_ recognition might result in the different substrate specificities of Prx and OxyR proteins.

### 3.6. NO induces intermolecular disulfide bonds with further oligomerization   

**Figure 6:**
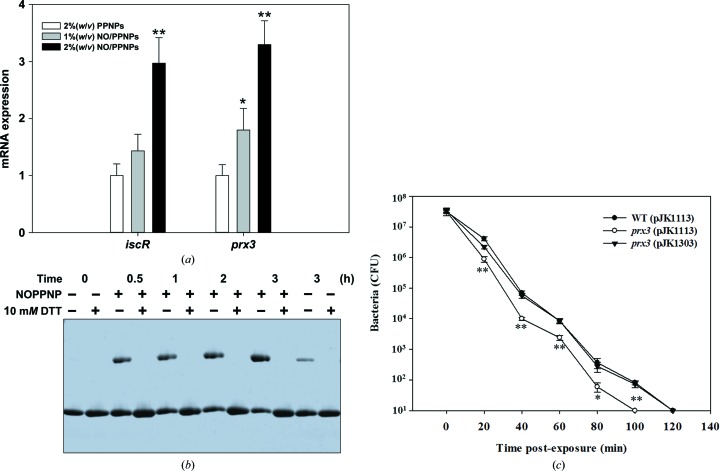
Effect of NO exposure on *Vvprx3* expression and *Vv*Prx3 protein. (*a*) Total RNA was isolated from wild-type cells grown aerobically to an *A*_600_ of 0.5 after exposure to various concentrations of PLGA–PEI nanoparticles (PPNPs) or NO/PPNPs for 20 min. PPNPs were used as blank nanoparticles. The *iscR* and *prx3* mRNA levels were determined by qRT-PCR analyses, and the *iscR* and *prx3* mRNA levels in the wild type exposed to 0.2%(*w*/*v*) PPNPs were set to 1. Error bars represent the standard deviation (SD). *, *P* < 0.05; **, *P* < 0.005. (*b*) *Vv*Prx3 (C73S) was incubated with NO/PPNPs for the times shown. A total of 3.3 µ*M* NO accumulated in the reaction mixture per hour. The reaction was terminated by adding 2.5 m*M* iodoacetamide to prevent nonspecific disulfide bonds. Aliquots were taken from the reaction mixture and loaded onto SDS–PAGE under reducing conditions (DTT +) or nonreducing conditions (DTT −). (*c*) Survival of *V. vulnificus* strains in the presence of NO stress. Wild-type bacteria (WT) or *prx3*-deleted bacteria (*prx3*) were transformed with the empty expression vector pJK1113 or the functional *prx3*-containing vector pJK1303. Well grown bacterial cells were exposed to NO/PPNPs for the times shown and viable cells were counted as colony-forming units (CFU) on LBS agar plates. Error bars represent the standard deviation (SD). *, *P* < 0.05; **, *P* < 0.005.

Increasing attention has been given to the NO-mediated reactions of host immune systems (Bogdan *et al.*, 2000 ▸; Nathan & Shiloh, 2000 ▸). A large amount of NO is synthesized in macrophages or epithelial cells stimulated by pathogens or by substances secreted by pathogens (Beckman & Koppenol, 1996 ▸; Fang, 2004 ▸). To investigate the functional relevance of *Vv*Prx3 and NO, we examined whether NO can induce the disulfide bonding of *Vv*Prx3, similar to H_2_O_2_ (Engelman *et al.*, 2013 ▸). Although Prxs are known to scavenge reactive nitrogen species such as the peroxinitrite that is rapidly generated by combining NO and superoxide ion (Fang, 2004 ▸; Beckman & Koppenol, 1996 ▸; Pfeiffer *et al.*, 1997 ▸), direct treatment with NO has not been tested on Prxs. We treated *V. vulnificus* with NO-releasing polymeric nanoparticles (NO/PPNPs), which have recently been developed to allow the sustained release of NO in solution (Nurhasni *et al.*, 2015 ▸), and then measured the mRNA levels of *prx3* and its known transcription factor *iscR*. The results showed that the mRNA levels of *prx3* and *iscR* significantly increased, implying that NO induces *Vv*Prx3 in *V. vulnificus via* IscR (Fig. 6 ▸ *a*).

We next tested whether NO directly induces disulfide bonds in the *Vv*Prx3 protein. NO/PPNPs were treated with *Vv*Prx3 protein, and the disulfide-bond formation of the protein was analyzed by SDS–PAGE and size-exclusion chromatography (Fig. 6 ▸ *b* and Supplementary Fig. S7). Compared with air-oxidized *Vv*Prx3 protein, significant protein bands representing the disulfide bond-linked *Vv*Prx3 increased on the SDS–polyacrylamide gel under nonreducing conditions after treatment of the nanoparticles releasing ∼1 µ*M* NO. The NO concentration in solution is much lower than the effective concentration (∼10 µ*M*) of H_2_O_2_ for *Vv*Prx3 (Fig. 6 ▸ *b*). Thus, our results suggest that *Vv*Prx3 could respond to NO stress through a similar mechanism as for H_2_O_2_ stress.

### 3.7. *Vv*Prx3 supports the survival of *V. vulnificus* under NO stress   

To evaluate the role of *Vv*Prx3 in resistance to NO in bacteria, the survival rates of wild-type and *prx3* gene-deleted *V. vulnificus* strains were measured. Compared with the wild-type strain, the *Prx3* mutant strain exhibited substantially impaired growth in a minimal medium containing NO/PPNPs. When the functional *prx3* gene (pJK1303) was added back to the mutant strain, the bacterial survival rate was restored (Fig. 6 ▸ *c*). Taken together, our results suggest that *Vv*Prx3 plays an important role in scavenging NO imposed on the bacteria.

## 4. Discussion   

Crystal structures of *Vv*Prx3, a bacterial 1-Cys Prx, were determined in different redox states in this study. The H_2_O_2_ binding site near the C_P_ residue was also shown. Although structural and sequence similarities to the 1-Cys Prx AhpE from *M. tuberculosis* were identified, especially in the dimeric assembly with the A-type interface, our structures provide unexpected and important insights into the function and molecular mechanism of Prxs. One striking feature of oxidized Prx3 (C73S) is a dimeric C-type interface with structural changes, which was previously observed in the oxidized form of *Hs*Prx5 belonging to the atypical 2-Cys Prx family (Smeets *et al.*, 2008 ▸). Since the C-type interface has been observed in two different kinds of Prxs, this oligomeric interface could be shared by many other Prx proteins. This study further observed that protein–protein interaction at the C-type interface was induced by both NO and peroxides. Owing to the oxidation-dependent C-type interface, *Vv*Prx3 formed a higher order linear oligomer in response to peroxides and NO. This linear oligomeric structure is distinct from the circular decameric assembly of typical 2-Cys Prxs, which is generated by alternate A-type and B-type interfaces. Since the linear oligomer of *Vv*Prx3 is generated only in the oxidized state, the redox-dependent oligomerization of *Vv*Prx3 differs from that of typical 2-Cys Prxs. Interestingly, this linear oligomeric form was also predicted in *Hs*Prx5 (Smeets *et al.*, 2008 ▸).

**Figure 7:**
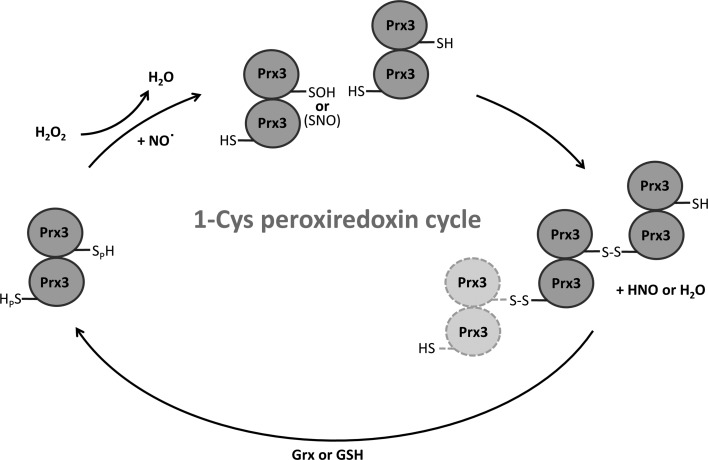
Proposed catalytic cycle of 1-Cys *Vv*Prx3. Each Prx3 polypeptide is represented by grey circles, with the peroxidatic Cys48 highlighted according to its redox state: sulfhydryl, –SH; sulfenic acid, –SOH; nitrosothiol, –SNO; disulfide, –S—S–. Potential conformations of additional dimers are indicated by light grey ovals.

According to the prevailing mechanisms, the C_P_ in 1-Cys Prxs is itself oxidized to C_P_-SOH through attack of the substrate peroxide. C_P_-SOH is then reduced by the reductase Grx or GSH (Noguera-Mazon *et al.*, 2006 ▸; Kang *et al.*, 1998 ▸; Rhee *et al.*, 2005 ▸). This study presents another pathway for the reduction of C_P_-SOH in 1-Cys Prxs, which might complement the direct reduction of C_P_-SOH by Grx or GSH under certain circumstances. In this pathway, the catalytic cysteine residue (C_P_) in 1-Cys Prx plays a dual role in the catalytic cycle. Not all *Vv*Prx3 proteins are simultaneously oxidized by H_2_O_2_. Thus, remaining or unreacted *Vv*Prx3 could act on other *Vv*Prx3s with C_P_-SOH. The C_P_-SOH can be reduced and resolved by free C_P_ in a different dimer of *Vv*Prx3, in which the free C_P_ acts as a C_R_. The complementary structural features at the C-type interface provide specific interactions with *Vv*Prx3 proteins, facilitating the prompt formation of a disulfide bond between C_P_-SOH and the free C_P_ of *Vv*Prx3 proteins (Fig. 1 ▸ *c*). This reaction could also occur successively, leading to the formation of the linear oligomers observed in the crystal structure (Supplementary Fig. S4). Finally, cellular GSH or Grx that is reduced by GSH reduces the disulfide bond in the oligomer, as proposed previously (Kim *et al.*, 2014 ▸). NO can be held in the H_2_O_2_ binding site of the *Vv*Prx3 active site to be readily attacked by C_P_. The free C_P_ from another *Vv*Prx3 molecule resolves the resulting nitrosyl­ated cysteine (C_P_-NO), as depicted in Fig. 7 ▸. Many different members are present in the 1-Cys Prx family. Of these, 1-Cys Prxs from yeast have previously been characterized. As in *Vv*Prx3, the intermolecular disulfide bond between C_P_ was observed in yeast 1-Cys Prx on an SDS–polyacrylamide gel under non­reducing conditions and predicted a doughnut-like oligomer formation in the oxidized state (Pedrajas *et al.*, 2016 ▸). We speculate that yeast 1-Cys Prx might share a catalytic cycle with *Vv*Prx3, although further studies are required to elucidate its mechanism.

The pathogenic bacterium *V. vulnificus* can defend against NO stress to increase survival in this harsh host environment. A previous study showed that *Vv*Prx3 is important in the pathogenicity of *V. vulnificus* in mice (Lim *et al.*, 2014 ▸). In this study, we observed that NO induced *prx3* together with *iscR* in *V. vulnificus* and that NO is a substrate of *Vv*Prx3. These findings suggest that *Vv*Prx3 is important for bacterial survival in NO-challenged environments and might account for the involvement of *Vv*Prx3 in pathogenicity in the host.

Diverse peroxiredoxins exist in nature and play different roles in the scavenging of toxic radicals. We have proposed a molecular mechanism for bacterial 1-Cys Prxs and a new role in scavenging NO in this study, which may in part account for the pathogenesis of *V. vulnificus*. Our findings expand the understanding of the molecular mechanism of survival of pathogenic bacteria in the host environment by scavenging diverse oxidative and nitrosative stresses.

## Supplementary Material

PDB reference: reduced Prx3 (C48D/C73S), 5k1g

PDB reference: oxidized Prx3 (C73S), 5k2i

PDB reference: H_2_O_2_-bound Prx3 (C48D/C73S), 5k2j

Supplementary Tables and Figures.. DOI: 10.1107/S205225251701750X/lz5017sup1.pdf

## Figures and Tables

**Table 1:** Data-collection and refinement statistics Values in parentheses are for the highest resolution shell.

	Oxidized Prx3 (C73S)	Reduced Prx3 (C48D/C73S)	H_2_O_2_-bound Prx3 (C48D/C73S)
Data collection			
Space group	*P*2_1_2_1_2_1_	*P*3_2_21	*P*1
*a*, *b*, *c* (Å)	39.5, 57.4, 124	73.9, 73.9, 62.3	75.1, 97.7, 97.5
α, β, γ (°)	90, 90, 90	90, 90, 120	78.7, 67.3, 67.3
Resolution (Å)	50–1.48 (1.51–1.48)	50–1.90 (1.93–1.90)	50–1.90 (1.93–1.90)
*R*_merge_	0.07 (0.28)	0.06 (0.39)	0.06 (0.36)
〈*I*/σ(*I*)〉	25.9 (4.96)	39.0 (4.57)	14.5 (2.07)
Completeness (%)	98.6 (95.1)	99.3 (99.5)	90.0 (82.6)
Multiplicity	10.0 (6.0)	13.3 (7.1)	3.4 (2.2)
Refinement			
Resolution (Å)	18.9–1.48 (1.52–1.48)	32.0–1.90 (1.96–1.89)	19.7–1.91 (1.93–1.91)
No. of reflections	47559	14790	137002
*R*_work_/*R*_free_	0.181/0.198	0.220/0.258	0.195/0.244
Total No. of atoms	2568	1189	14956
No. of ligands	0	0	4
No. of water molecules	231	37	805
Wilson *B* factor (Å^2^)	12.1	15.7	17.3
R.m.s. deviations			
Bond lengths (Å)	0.007	0.002	0.003
Bond angles (°)	1.1	0.57	0.57
Ramachandran plot			
Favoured (%)	95.5	94.7	93.4
Allowed (%)	4.5	5.3	6.5
Outliers (%)	0.0	0.0	0.1
Molecules in asymmetric unit	2	1	12
PDB code	5k2i	5k1g	5k2j

The *R*_free_ value was calculated using a test-set size of 9.95%.
