# Supplementary material for: Crystal structure of peroxiredoxin 3 from Vibrio vulnificus and its implications for scavenging peroxides and nitric oxide
Source: IUCrJ. 2018 Jan 1;5(Pt 1):82–92. doi: 10.1107/S205225251701750X (PMC5755580; doi:10.1107/S205225251701750X)
Supplement: Supplementary file 1 [file m-05-00082-sup1.pdf]

IUCrJ

Volume 5 (2018)

Supporting information for article:

Crystal structure of peroxiredoxin 3 from *Vibrio vulnificus* and its implications for scavenging peroxides and nitric oxide

Jinsook Ahn, Kyung Ku Jang, Inseong Jo, Nurhasni Hasan, Jin-Wook Yoo, Sang Ho Choi and Nam-Chul Ha

Table S1 Plasmids and strains used in this study

Strain or plasmid	Relevant characteristics[a]	Reference or source
**Bacterial strains**		
V. vulnificus		
MO6-24/O	Clinical isolate; virulent	Wright *et al*., 1990
OH0701	MO6-24/O with *prx1*::*nptI*; Km^r	Baek *et al*., 2009
OH0505	MO6-24/O with *prx2*::*nptI*; Km^r	Oh *et al*., 2008
JK134	MO6-24/O with Δ*prx*3	Lim *et al*., 2014
E. coli		
S17-1λ*pir*	λ-*pir* lysogen; *thi pro hsdR hsdM*$^+$ *recA* RP4-2 Tc::Mu-Km::Tn7;Tp^r Sm^r; host for π-requiring plasmids; conjugal donor	Simon *et al*., 1983
**Plasmids**		
pJK1113	pKS1101 with *nptI*; Ap^r, Km^r	Lim *et al*., 2014
pJK1303	pJK1113 with *prx*3; Ap^r, Km^r	Lim *et al*., 2014

[a] Tp^r, trimethoprim-resistant; Sm^r, streptomycin-resistant; Ap^r, ampicillin-resistant; Km^r, kanamycin-resistant

Table S2 Oligonucleotides used in this study

Name	Oligonucleotide sequence (5' → 3')[a]	Use
For qRT-PCR		
ISCR_qRT_F	GATATGCGGTAACGGCAATGCT	Quantification of *iscR* expression
ISCR_qRT_R	TAAGAGAGCGAAATCCCCTGACG	
PRX3_qRT_F	TGAAAGCCTGGGGTGAAGCA	Quantification of *prx3* expression
PRX3-qRT_R	ATCGCGTAGCGTTGAGAGCG	
16S-qRT-F	CGGCAGCACAGAGAAACTTG	Quantification of the16S rRNA expression
16S-qRT-R	CCGTAGGCATCATGCGGTAT	

[a]The oligonucleotides were designed using the *V. vulnificus* MO6-24/O genomic sequence (GenBank™ accession number CP002469 and CP002470, www.ncbi.nlm.nih.gov).

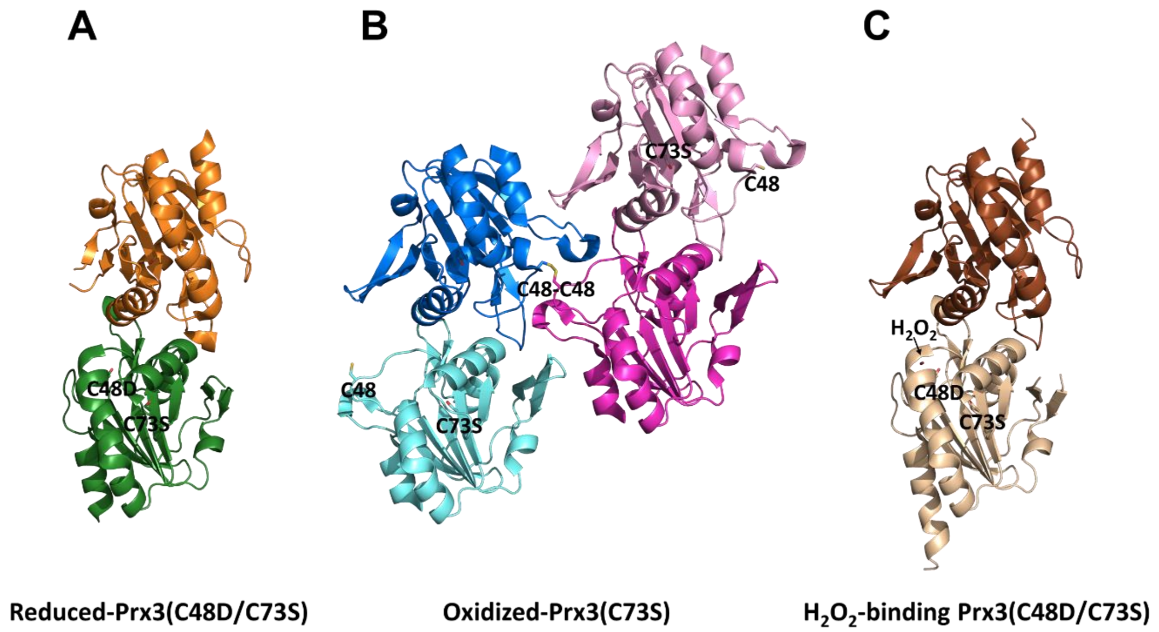

Figure S1 Dimeric interfaces of *Vv*Prx3 structures. Dimeric interfaces of the structures in reduced-Prx3 (C48D/C73S) (A), oxidized-Prx3 (C73S) (B) and H_2O_2-binding Prx3 (C48D/C73S) (C) are shown as ribbon diagram. Each protomers are drawn in different colors, and the side chains of Cys48 and Ser73 are displayed. The black arrow indicates H_2O_2.

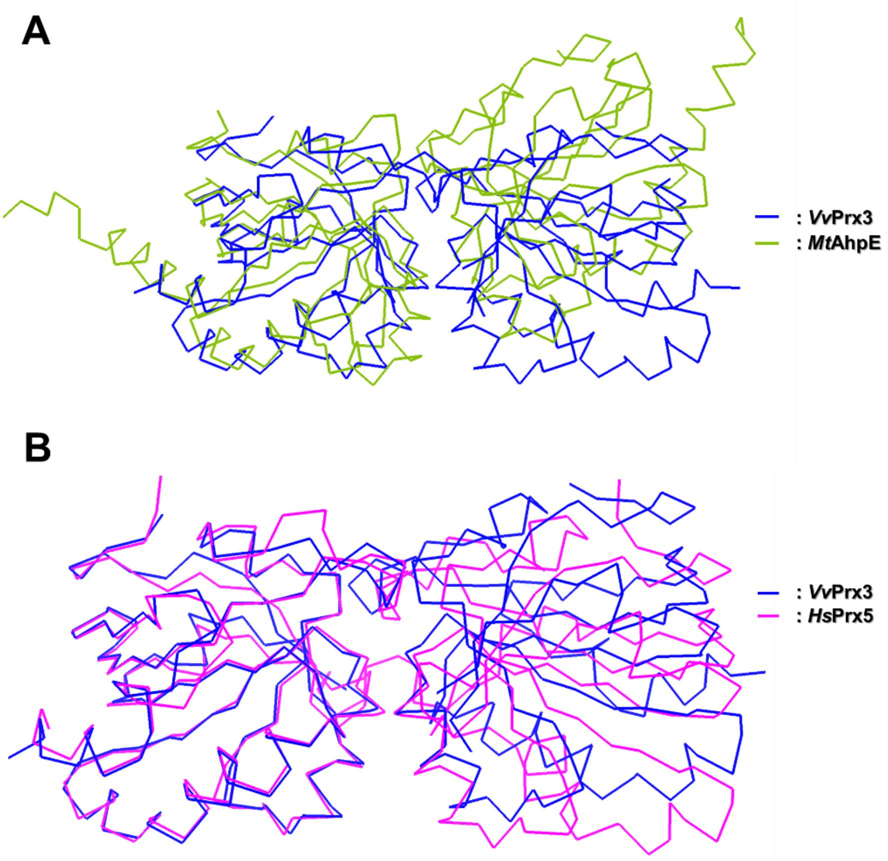


```
VvPrx3  ----------------MI--AQGQTLPNAT------------LSQLTKEGMVHHPV----------------------LE : 28
MtAhpE  ----------------MLN--VGATAPDFT------------LRDQNQQLVTLRGYR----------------------- : 27
HsPrx5  MGLAGVCALRRSAGYILVGGAGGQSAAAAARRCSEGEWASGGVRSFSRAAAAMAPIKVGDAIPAVEVFEGEPGNKVNLAE : 80

                           Cp                       C2
VvPrx3  LFAGKKVVLFAVPGAFTPTCSEAHLPGYIVLADQLKAKGVDLIACVSVNDAFVMKAWGEAQNAE-EILMLADGDASFTKA : 107
MtAhpE  --GAKNVLLVFFPLAFTGICQG-ELDQLRDHLPEFEND-DSAALAISVGPPPTHKIWATQSGFTFPLLSDFWPHGAVSQA : 103
HsPrx5  LFKGKKGVLFGVPGAFTPGCSKTHLPGFVEQAEALKAKGVQVVACLSVNDAFVTGEWGRAHKAEGKVRLLADPTGAFGKE : 160

                                                    CR
VvPrx3  LGLEMDT---AGFGGLRSQRYAMIIDNGVVTTLNVEAPKS-FEVSNAETILAAL-- : 157
MtAhpE  YGVFNEQ------AGIANRGTFVVDRSGIIRFAEMKQPGEVRDQRLWTDALAALTA : 153
HsPrx5  TDLLLDDSLVSIFGNRRLKRFSMVVQDGIVKALNVEPDGTGLTCSLAPNIISQL-- : 214
```

Figure S2 Structural superposition of *Vv*Prx3 with homologous proteins. (A) *Vv*Prx3 (C48D/C73S) (blue) is superposed onto AhpE from *M. tuberculosis* (green; PDB code 1XXU). (B) Dimeric unit of *Vv*Prx3 (C48D/C73S) structure in the reduced state (blue) is superposed on human PrxV (magenta; PDB code 1OC3), an atypical 2-Cys Prx. (C) The amino acid sequences comparison of *Vv*Prx3, AhpE from *M. tuberculosis*, and human PrxV. The positions of the peroxidatic (C_P), second (C_2), and resolving cysteine (C_R) are displayed above the sequences.

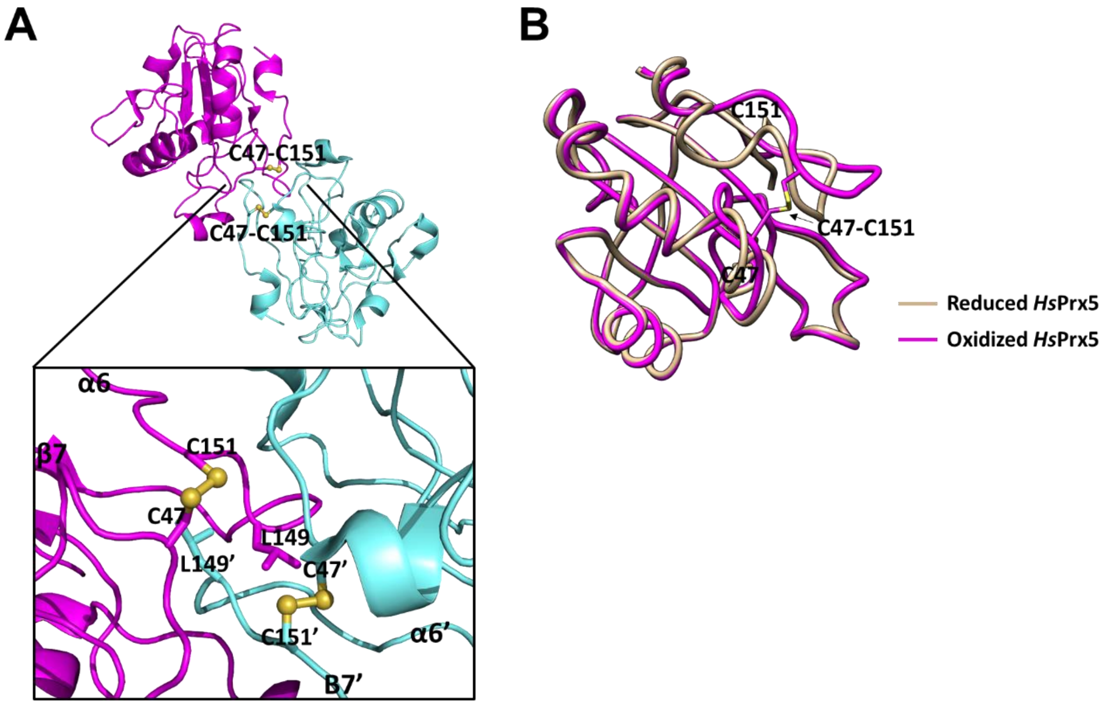

Figure S3 Structural comparison of *Hs*Prx5 between reduced and oxidized states. (A) A magnified view focusing on interactions at the C-type interface present in the intramolecular disulfide bond between Cys47 and Cys151 residues. The side-chains participated in the C-type interfaces are represented as balls and sticks. The labels of protomers are distinguished by ’. The two protomers are colored differently (magenta and cyan). (B) Structural superposition of *Hs*Prx5 in the reduced form (wheat) and oxidized form (magenta).

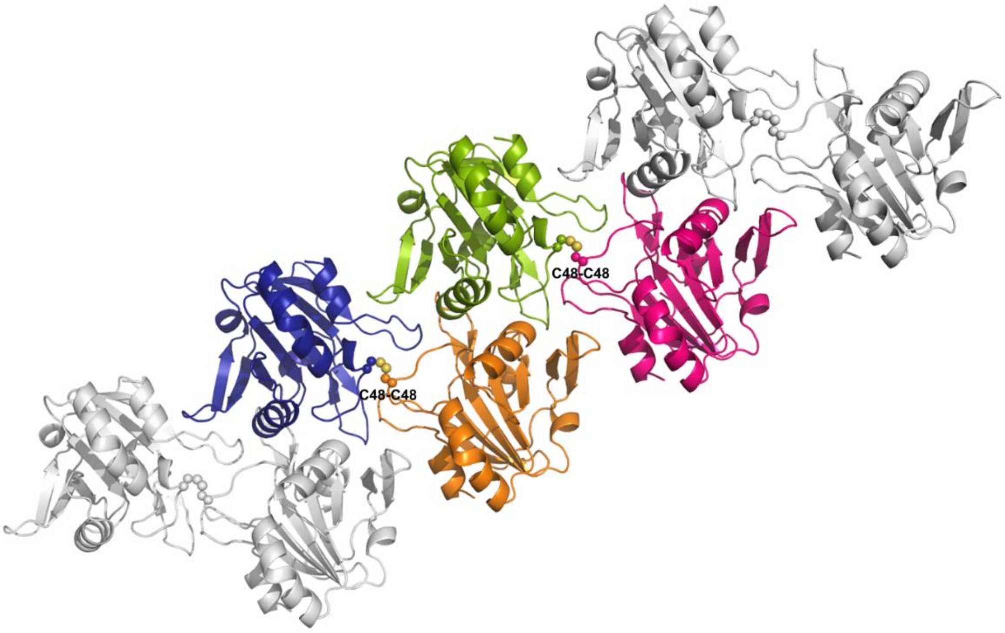

Figure S4 Oligomeric assembly observed in *Vv*Prx3 (C73S) structure in the disulfide state in the crystal. The asymmetric unit is shown in blue, orange, green, or magenta, while adjacent molecules are in gray. Each protomer is connected via disulfide bonds between Cys48s (balls).

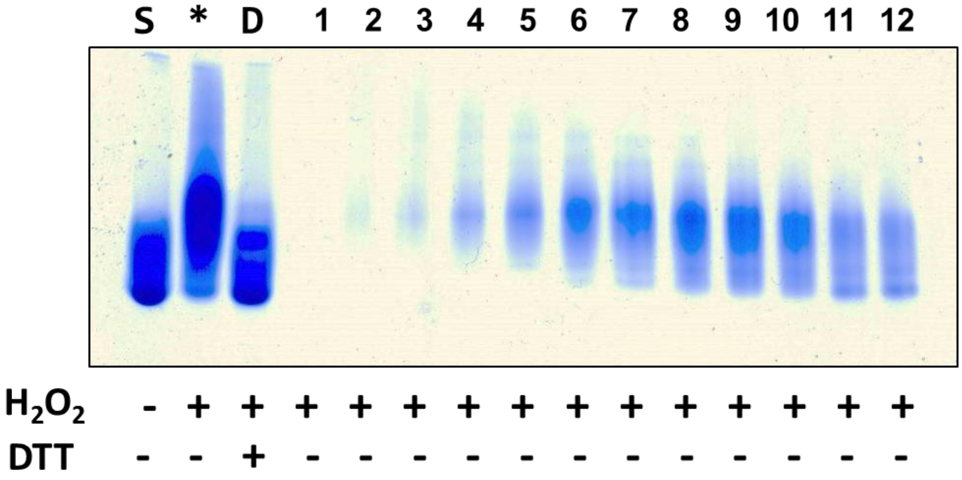

Figure S5 Native gel analysis of fractions of Fig. 4. Each fraction of the size exclusion chromatography in Fig. 4 was analyzed by native gel electrophoresis (*lanes 1-12*). The protein sample of *Vv*Prx3 (C73S) (*lane S*) was treated with 15 µM H_2O_2 for 60 min (*lane* *) and then 10 mM DTT (*lane D*). The protein sample treated with H_2O_2 was loaded onto the size exclusion chromatographic column (*lanes 1-12*).

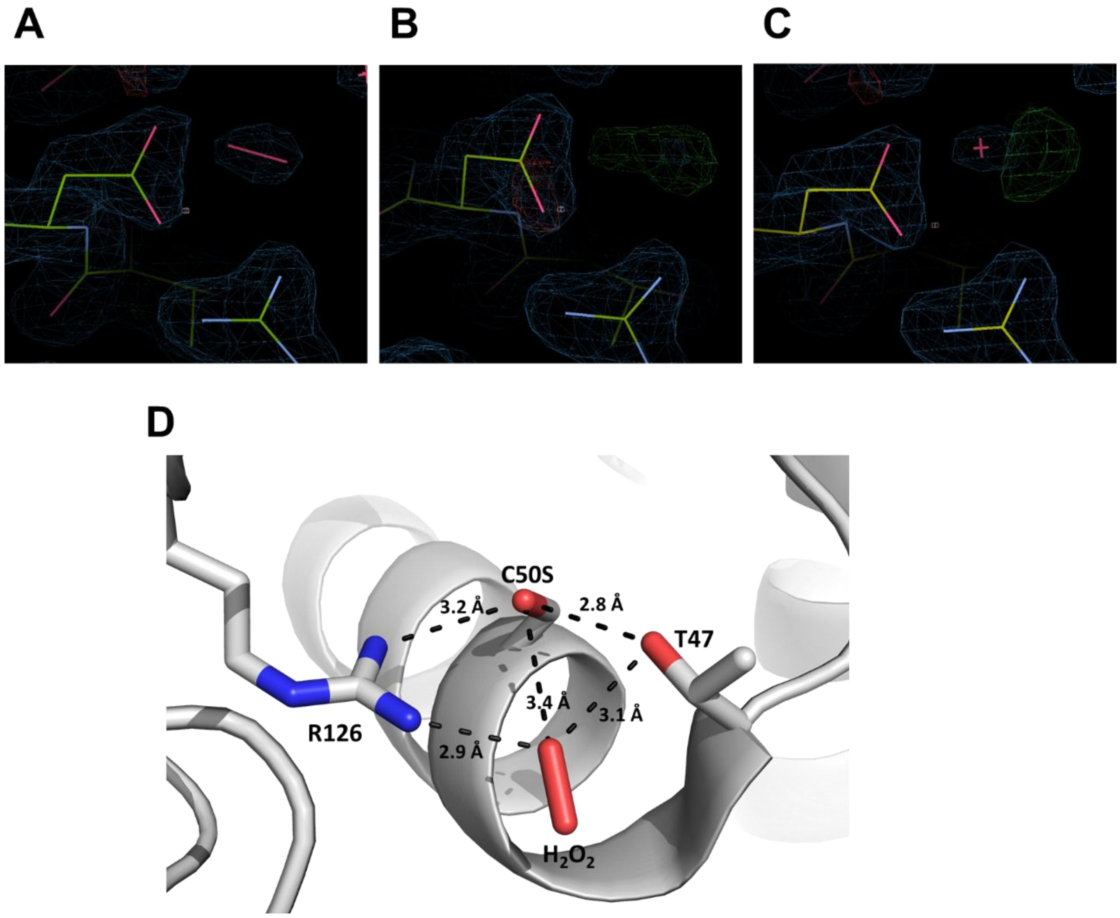

Figure S6 Electron density map of the H_2O_2-binding site of *Vv*Prx3 and *Ap*TPx. One H_2O_2 molecule was fitted to the electron density map (A). The electron density maps were refined with no molecule is assigned (B) and one water molecule is assigned (C). 2Fo − Fc and Fo − Fc electron density maps are represented as blue and green, respectively. (D) The H_2O_2 binding site of thioredoxin peroxidase from *Aeropyrum pernix* K1 (PDB code 3A2W). Broken lines indicate interactions between residues and H_2O_2.

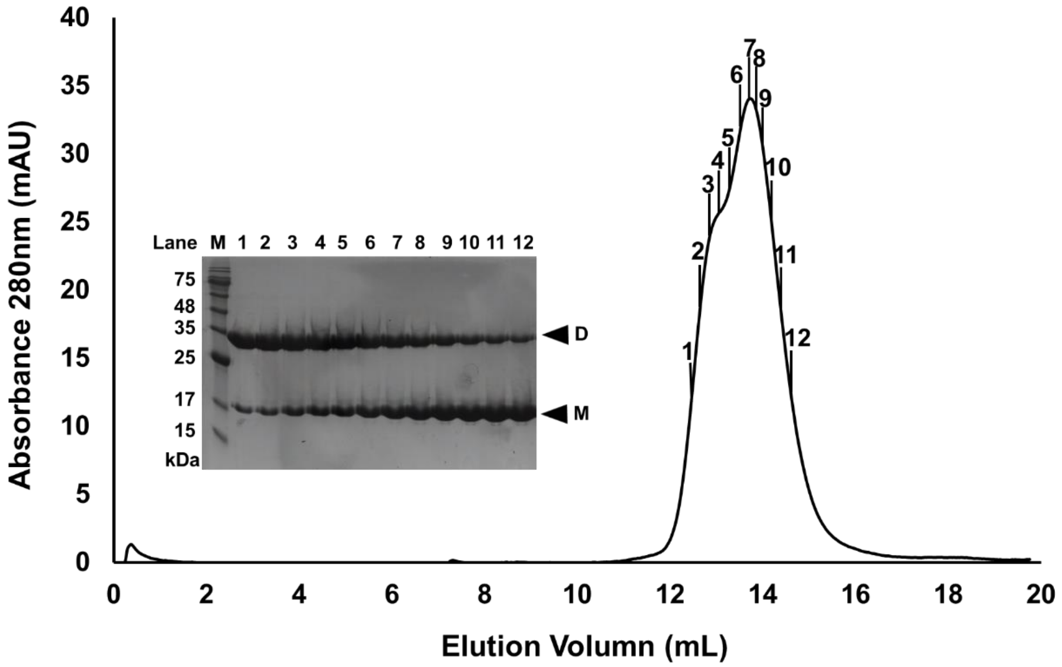

Figure S7 Oligomerization states of *Vv*Prx3 in solution after treatment with NO-releasing nanoparticle (1 mg) for 30 min. Oligomeric states were analyzed using size exclusion chromatography combined with SDS-PAGE under non-reducing conditions. The protein band of disulfide bond-containing *Vv*Prx3 (*D*) and bands that lack a disulfide bond (*M*) are indicated.

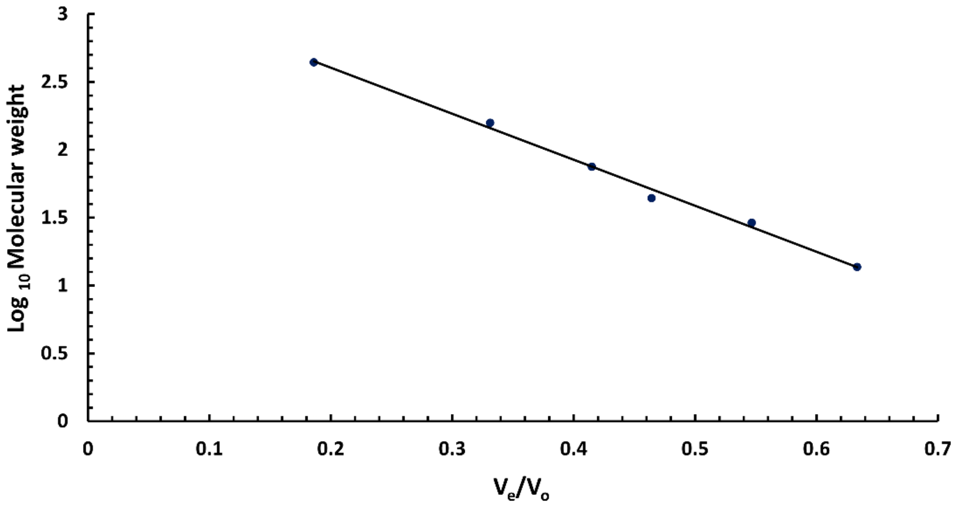

Figure S8 Standard curve of analytic size exclusion chromatography. 11.54 ml: ferritin (440 kDa), 13.79 ml: aldolase (158 kDa), 15.09 ml: conalbumin (75 kDa), 15.8 ml: ovalbumin (43 kDa), 17.12 ml: carbonic anhydrase (29 kDa), 18.54 ml: ribonuclease A (13.7 kDa), 18.66 ml: ribonuclease A (13.7 kDa). V_e indicates elution volume and V_o indicates void volume.
